# Visualization and Comparison of the Level of Apurinic/Apyrimidinic Endonuclease 1 in Live Normal/Cancerous and Neuron Cells with a Fluorescent Nanoprobe

**DOI:** 10.3390/molecules28093935

**Published:** 2023-05-07

**Authors:** Peng Lu, Xiangjian Cao, Jinghui Zheng, Ying Sun, Ziyu Tang, Meiping Zhao

**Affiliations:** 1 Beijing National Laboratory for Molecular Sciences and MOE Key Laboratory of Bioorganic Chemistry and Molecular Engineering, College of Chemistry and Molecular Engineering, Peking University, Beijing 100871, China; 2 Institute of Food Science and Technology, Chinese Academy of Agricultural Sciences, Beijing 100193, China

**Keywords:** apurinic/apyrimidinic endonuclease 1 (APE1), fluorescent nanoprobe, live cell fluorescence imaging, human normal breast epithelial cells (MCF-10A), human breast adenocarcinoma cells (MCF-7)

## Abstract

As a major apurinic/apyrimidinic endonuclease and a redox signaling protein in human cells, APE1 plays a crucial role in cellular function and survival. The relationship between alterations of APE1 expression and subcellular localization and the initiation, development and treatment of various cancers has received extensive attention. However, comparing the in-vivo activity of APE1 in normal and cancerous breast live cells remains challenging due to the low efficiency of commonly used liposome transfection methods in delivering DNA substrate probes into human normal breast epithelial cells (MCF-10A). In this work, we develop a DNA/RNA hybrid-based small magnetic fluorescent nanoprobe (25 ± 3 nm) that can be taken up by various live cells under magnetic transfection. The D_0_/R-nanoprobe demonstrates an outstanding specificity toward APE1 and strong resistance to the cellular background interference. Using this nanoprobe, we are not only able to visualize the intracellular activity of APE1 in breast ductal carcinoma (MCF-7) live cells, but also demonstrate the APE1 activity in MCF-10A live cells for the first time. The method is then extended to observe the changes in APE1 levels in highly metabolically active neuroendocrine cells under normal conditions and severe attacks by reactive oxygen species in real-time. The fluorescent nanoprobe provides a useful tool for studying the dynamic changes of intracellular APE1 in normal or cancerous live cells. It also displays the potential for visible and controllable release of miRNA drugs within live cells for therapeutic purposes.

## 1. Introduction

Human apurinic/apyrimidinic endonuclease 1 (APE1) is a multifunctional cellular protein that not only functions as a DNA repair enzyme but also as a redox regulator of transcription factors activation [1,2]. An abnormal expression and subcellular localization of APE1 have been observed in numerous cancers, neurological disorders and other diseases [3,4,5]. Given the critical role of APE1 in cellular function and survival, the correlation between alterations in APE1 expression levels and cytoplasmic/nuclear distribution and the onset, progression and treatment of various cancers has been extensively investigated [4,5,6,7,8]. Previous studies have reported that elevated levels of APE1 are associated with tumorigenesis, cancer aggressiveness, radio/chemotherapeutic resistance and a poor prognosis in several types of cancer [9,10,11]. Lowering APE1 levels have been shown to increase the sensitivity of certain cancers to platinating agents. Conversely, increasing the expression of APE1 can help to reduce the neurotoxicity caused by anticancer treatment [12].

In addition to a significantly elevated expression of APE1 in human breast carcinomas, a predominant cytoplasmic localization of APE1 has been observed in both lactating breast and breast carcinomas by immunohistochemistry [10]. Subsequent studies using in-vitro quantitative measurements such as LC/MS/MS or fluorescent probe-based enzymatic assays have revealed extremely high levels of APE1 in the nucleus of human breast adenocarcinoma cells (MCF-7) [13,14]. In contrast, the activity of APE1 in the cytoplasmic extracts of MCF-7 cells has been found to be as similarly low as in those of human normal breast epithelial cells (MCF-10A) based on fluorescent probe measurements [14]. These findings emphasize the importance of in-vivo comparisons of APE1 expression and localization in normal and cancerous breast live cells.

With the help of liposome transfection or other probe delivery methods, researchers have extensively studied the intracellular distribution of APE1 in the cytoplasm of MCF-7 and some other cell types. However, direct visualization of the intracellular APE1 activity in normal breast cells remains challenging due to the low uptake efficiency of external substrate probes by MCF-10A live cells and the limitation of specificity of many existing APE1 probes. Additionally, the variation of APE1 levels in live breast cells and neuron cells under different conditions has been much less explored.

In our previous work, we used avidin-modified Fe_3_O_4_@SiO_2_ magnetic nanoparticles (SiMNPs, ~67 nm) to transfer dsDNA probes into human cervical carcinoma (HeLa) cells [15]. More recently, we developed a DNA/RNA hybrid fluorescent probe that allowed for highly specific quantification of APE1 levels in different subcellular compartments in MCF-7, MCF-10A, and other cancerous or neuron-like (PC-12) cells [14]. In this study, we attempt to employ smaller magnetic nanoparticles (20~30 nm) to transfer the DNA/RNA hybrid fluorescent probes into normal/cancerous breast epithelial cells and neuron-like cells for in-vivo comparison. Our D_0_/R-nanoprobe shows outstanding specificity toward APE1 and strong resistance to cellular background interferences. We disclose for the first time the intracellular activity of APE1 in the cytoplasm of MCF-10A live cells. We also demonstrate the dynamic changes of APE1 level in PC-12 neuroendocrine cells under severe attack by reactive oxygen species (ROS) in real time. Our DNA/RNA hybrid fluorescent nanoprobe provides a useful tool for continuously monitoring the variation of intracellular APE1 in normal or cancerous live cells. It also holds potential for the visible and controllable release of miRNA drugs within live cells for therapeutic purposes.

## 2. Results and Discussion

### 2.1. Construction and Characterization of the DNA/RNA Hybrid Fluorescent Nanoprobe (D_0_/R-Nanoprobe)

**Figure 1:**
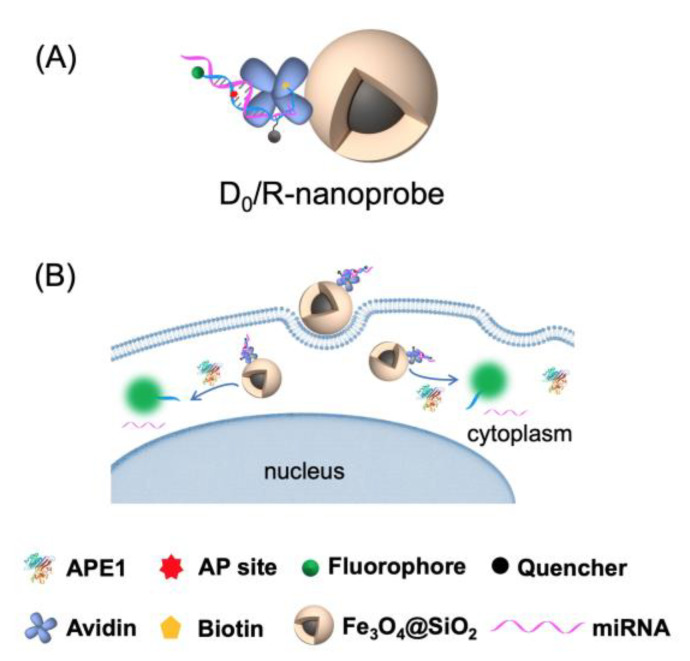
(**A**) Schematic representation of the AP site-containing DNA/RNA hybrid (D_0_/R) nanoprobe; (**B**) Fluorescence imaging of APE1 activity in live cells by using the D_0_/R-nanoprobe.

In our previous study, we utilized avidin-modified SiMNPs to deliver dsDNA probes into HeLa cells [15]. Recently, we have developed a more specific DNA/RNA hybrid fluorescent probe allows for high-throughput quantification of APE1 levels in subcellular compartments of various cell types [14]. To create a fluorescent nanoprobe with both guaranteed specificity for APE1 and high uptake efficiency across different cell types, we firstly synthesized a 3′ biotin-tagged, uracil-containing ssDNA labeled with FAM/BHQ1 (Table S1, D_U_-P). After treatment with uracil-DNA glycocasylase (UDG), the resulting biotin-labeled AP site-containing ssDNA (D_0_-P) was attached to avidin-modified SiMNPs with an average diameter of ~22 nm. We then added RNA strands (R, Table S1) to obtain the D_0_/R-nanoprobe (Figure 1A). The zeta potential values of the nanoparticles, as shown in Figure S1, confirm the avidin modification on the surface of the SiMNPs and the attachment of the DNA/RNA hybrid probes to the avidin-modified SiMNPs. The transmission electron microscopy (TEM) analysis demonstrates that the D_0_/R-nanoprobes have an overall size of 25 ± 3 nm (Figure S2). The dynamic light scattering (DLS) measurement results indicate that the D_0_/R-nanoprobes are also well dispersed with an average hydrodynamic size of 246 ± 16 nm in 10 mM PBS solution (Table S3). Figure S3 demonstrates the rapid separation of the uniform dispersion of the D_0_/R-nanoprobes in the presence of an external magnetic field.

For comparison, the biotin-labeled uracil-containing ssDNA without UDG pretreatment was also attached to the SiMNPs. After annealing to the RNA strand, the resulting D_U_/R-control nanoprobe was used as a reference probe to monitor the potential UDG-APE1 dual enzymatic Base Excision Repair (BER) signal and other background signals within the cells.

**Figure 2:**
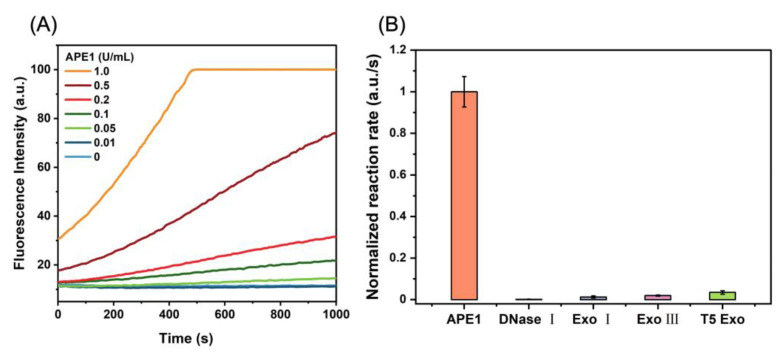
(**A**) Fluorescence curves of the reactions between D_0_/R-nanoprobe (100 μg/mL) and APE1 at different concentrations. (**B**) Selectivity of D_0_/R-nanoprobe (100 μg/mL) toward APE1 (1.0 U/mL) over other nucleases (DNase I: 1.0 U/mL; Exo I: 2.5 U/mL; Exo III: 20 U/mL; T5 Exo: 1.0 U/mL) in Buffer 1.1 + 0.02% Triton X-100. All experiments were repeated at least three times.

The D_0_/R-nanoprobe exhibits highly sensitive responses to APE1 within the concentration range of 0.01 to 1.0 U/mL, as demonstrated in Figure 2A and Figure S4. The detection limit is 0.005 U/mL. Based on the selectivity test results presented in Figure 2B, the discrimination capability of the D_0_/R-nanoprobe between APE1 and other enzymes far exceeds that of other existing fluorescent probes [14]. This superior selectivity can be attributed to the combination of the benefits derived from a DNA/RNA hybrid probe [14] and avidin-modified SiMNPs [15].

### 2.2. Fluorescence Imaging of APE1 Activity in Live MCF-7 and MCF-10A Cells

**Figure 3:**
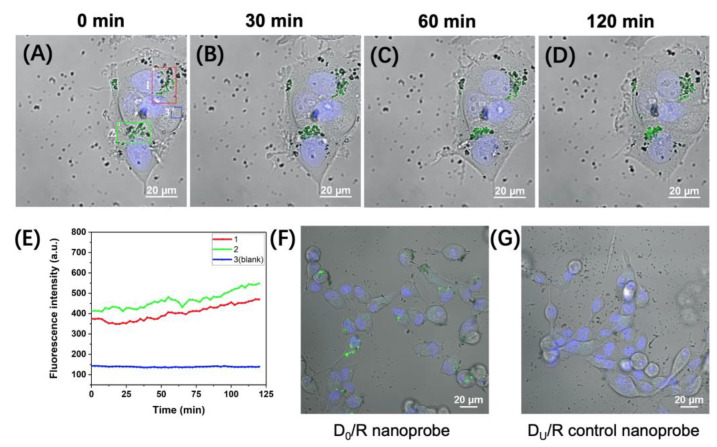
(**A**–**D**) Time-lapse fluorescence imaging of APE1 activity (green) in MCF-7 live cells after 2 h magnetic transfection of D_0_/R-nanoprobes (100 μg/mL). (**E**) Time courses of the fluorescence intensity of the three representative regions in MCF-7 live cells transfected with D_0_/R-nanoprobes. (**F**) Fluorescence imaging of APE1 activity (green) in MCF-10A live cells after 6 h magnetic transfection of D_0_/R-nanoprobes (100 μg/mL). (**G**) Fluorescence image of the MCF-10A live cells after 6 h magnetic transfection of D_U_/R-control nanoprobes (100 μg/mL, green). Hoechst 33342 was used to stain the nucleus (blue).

To monitor the intracellular activity of APE1 in real time, we delivered the D_0_/R-nanoprobes into MCF-7 live cells (Figure 1B). After magnetic transfection with 100 μg/mL D_0_/R-nanoprobes for 2 h, the probes were efficiently internalized by MCF-7 cells and emitted bright fluorescence signals in the cytoplasm (Figure 3A). Continuous imaging was performed over a period of 120 min, with images acquired at 30 min, 60 min and 120 min after the 2 h transfection shown in Figure 3B–D and Figure S5, respectively. Over time, the nanoprobes accumulate in the perinuclear region of cytoplasm and emit increasingly bright fluorescent signals. Due to their relatively large size (25 ± 3 nm) and spheric shape [16,17] the D_0_/R-nanoprobes are unable to passively diffuse through the nuclear pore and enter the nucleus.

To monitor changes in the fluorescence signals of the D_0_/R-nanoprobe in MCF-7 cells throughout the imaging period, we identified three representative regions (the brightest, less bright and background) in the field of view shown in Figure 3A–D, which are marked with boxes in different colors. In Regions 1 (red box) and -2 (green box), the bright fluorescence signals emitted by the distributed nanoprobes indicate relatively high levels of APE1 in the marked regions of the two cells. Region 3 (blue box) represents the background region where there are almost no nanoprobes. The time-dependent fluorescence intensity of the three selected regions was measured and plotted over the 120-min imaging period in Figure 3E. The fluorescence intensity of background Region 3 (blue curve) remains consistently low. In contrast, the fluorescence intensity of Regions 1 and 2 both continue to increase during the observation period, demonstrating the continuous response of the internalized D_0_/R-nanoprobes to changes in APE1 levels within the cytoplasm of the live cells.

Next, we attempted to visualize the intracellular activity of APE1 in MCF-10A live cells. After magnetic transfection of D_0_/R-nanoprobes following the same conditions as those used for MCF-7 cells (100 μg/mL, 2 h), no fluorescence signals are detected within the cells. These findings are apparently inconsistent with the in vitro measurement results that indicate comparable cytoplasmic APE1 levels in both MCF-7 and MCF-10A cells [14]. Further inspection on the cells using bright-field microscopy reveals that the uptake efficiency of the nanoprobes by MCF-10A is significantly lower than that observed in MCF-7 cells (Figure S6). Consequently, we extended the magnetic transfection time to 6 h and conducted experiments using both D_0_/R-nanoprobes and D_U_/R-control probes. Figure 3F and Figure S7 show bright fluorescence signals of D_0_/R-nanoprobes in the cytoplasm of MCF-10A cells. In contrast, the signals of D_U_/R-control probes in MCF-10A cells are much weaker (Figure 3G). We compared the relative increase of fluorescence intensity of the brightest fluorescence signals in the cells obtained by the two probes. The highest fluorescence intensity observed by the D_U_/R-control probe is close to the background signals obtained by D_0_/R-nanoprobes. We also tested incubating MCF-10A live cells with D_0_/R-nanoprobes or D_U_/R-control probes at a lower concentration (50 μg/mL, 6 h). Figure S8 shows that fewer fluorescence signals of D_0_/R-nanoprobes appear in the cytoplasm of MCF-10A cells compared with those in Figure 3F and Figure S7, while the fluorescence signals of D_U_/R-control probes are negligible under the same experimental conditions. These results confirm that the bright fluorescence signals emitted by D_0_/R-nanoprobes are generated by the cleavage of the AP sites by APE1 within the cells.

As the uptake efficiency of the nanoprobes by the two cell types varies significantly, it is challenging to make a quantitative comparison of the intracellular APE1 level between cancerous and normal human breast cells. Nonetheless, the results presented in Figure 3F validate that the cytoplasmic APE1 level in non-cancerous human breast cells is not significantly lower than that in the carcinoma cells. This finding holds great significance for the development and assessment of APE1-targeted anti-cancer drugs and therapies. Additionally, the established method provides a convenient tool for further investigation of the impact of estrogenic substances on the expression of APE1 in breast cell lines.

### 2.3. Time-Lapse Fluorescence Imaging of the APE1 Activity in PC-12 Live Cells with the D_0_/R-Nanoprobes

**Figure 4:**
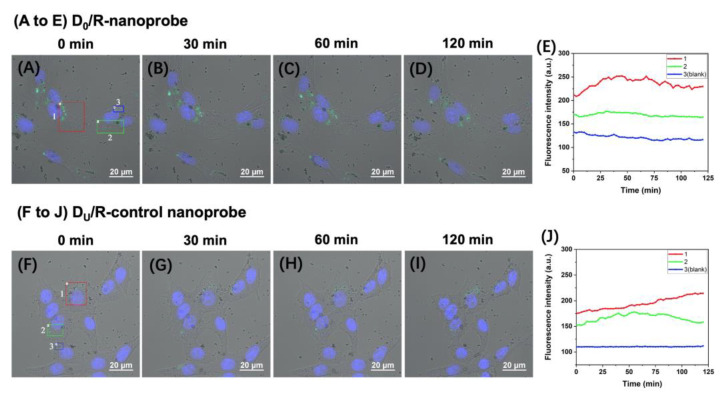
Time-lapse fluorescence imaging of the APE1 activity (green) in PC-12 live cells by using the D_0_/R-nanoprobes (**A**–**D**) and D_U_/R-control nanoprobes (**F**–**I**), respectively. (**E**) Time courses of the fluorescence intensity of the three representative regions in PC-12 live cells transfected with D_0_/R-nanoprobes. (**J**) Time courses of the fluorescence intensity of the three representative regions in PC-12 live cells transfected with D_U_/R-control nanoprobes. The cells were incubated with 100 μg/mL of the nanoprobes for 120 min and analyzed by confocal microscopy. Hoechst 33342 was used to stain the nucleus (blue).

PC-12 cells have been widely utilized as a model for studying neuron cells [18,19]. We incubated PC-12 cells with 100 μg/mL of D_0_/R-nanoprobes and captured time-lapse fluorescence images to monitor the activity of APE1 in the cells. Figure 4A–D display images taken at 0 min, 30 min, 60 min and 120 min after a 2-h magnetic transfection, respectively. Fluorescence signals are clearly visible in the cytoplasm of PC-12 cells, indicating the efficient uptake of nanoprobes by the cells. We conducted control experiments using D_U_/R-control nanoprobes in a same manner (Figure 4F–I), which only show faint fluorescence in some cells. These results confirm that the intracellular fluorescence signals observed in Figure 4A–D repredominantly generated by the cleavage of AP-sites in D_0_/R-nanoprobes by APE1.

We selected three representative regions marked with boxes in different colors in the field of view shown in Figure 4A–D to track the continuous variation of the fluorescence signals of the nanoprobes inside the PC-12 cells during the imaging period. The bright fluorescent signals in Regions 1 (red box) and 2 (green box) indicate relatively high APE1 levels in the marked regions of the two cells, while Region 3 (blue box) represents the background region where there are almost no fluorescent signals. The time-dependent fluorescence intensity of the three selected regions over the 120-min imaging period is measured and plotted in Figure 4E. The fluorescence intensity of the brightest region (Region 1, red box) increases along with time and reached a plateau after ~30 min (the red curve in Figure 4E). The detailed fluorescence images acquired at ~40 min after magnetic transfection area shown in Figure S9. The signals in Region 2 (green box, green curve) are relatively weak without significant fluctuations.

For comparison, we continuously monitored and analyzed the signals of the D_U_/R-control nanoprobe in PC-12 cells (Figure 4F–I). Figure 4J shows the time courses of the fluorescence signals of the control probe in the selected regions. The fluorescence intensity of the brightest region (red curve) relative to the background level (Region 3, blue curve) in Figure 4J is lower than that in Figure 4E. The gradually elevated signals might have been generated by the removal of the uracil in the probe by UDG in the cells [20] followed by cleavage of the resultant AP site by APE1. The difference between the fluorescence signals of the two nanoprobes proves the reliability of the results obtained by D_0_/R-nanoprobes within the live cells. Moreover, the fluorescence signals around the D_0_/R-nanoprobes do not diffuse quickly, as shown in Figure 4A–D, allowing precise localization of the occurrence of enzymatic reactions and continuous monitoring of the distribution of the signals under external stimulations.

Under identical transfection and observation conditions, the fluorescence intensity in the brightest region of PC-12 cells does not increase as significantly as in MCF-7 live cells. These findings highlight the importance of dynamically monitoring changes in APE1 levels across different types of live cells.

### 2.4. Real-Time Monitoring of the Changes of APE1 Level in PC-12 under Severe Attack by ROS

Neurons have a high oxygen consumption, which puts them at risk for high levels of oxidative DNA damage. In recent years, besides its DNA-repair function, numerous other biological roles of APE1 in the nervous system have been discovered. Tert-butyl hydroperoxide (t-BHP) is a widely used pro-oxidant agent that induces oxidative stress [21,22,23]. With increased treatment concentration and exposure time, t-BHP can lead to cell apoptosis or necroptosis [23]. Overexpression of APE1 has been shown to protect cells from oxidative DNA damage and apoptosis induced by ROS [7,12,24].

**Figure 5:**
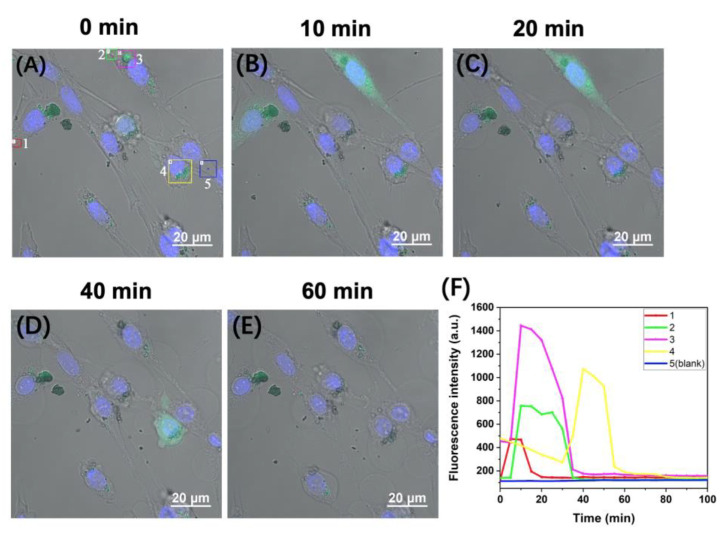
(**A**–**E**) Monitoring of the variation of APE1 activity in PC-12 live cells under the treatment with tert-butyl hydroperoxide (tBHP). (**F**) Time courses of the fluorescence intensity of the five representative regions in PC-12 live cells transfected with D_0_/R-nanoprobes. The cells were incubated with 100 μg/mL D_0_/R-nanoprobe for 120 min. tBHP was added to the imaging medium at a concentration of 2.5 mM after the magnetic transfection of D_0_/R-nanoprobe. Hoechst 33342 was used to stain the nucleus (blue).

To track changes in APE1 levels in live neuron-like cells under ROS attack, we transfected PC-12 cells with D_0_/R-nanoprobes and performed time-lapse imaging while treating the cells with tBHP. The cells successively emit very bright fluorescence throughout the whole cell, indicating a dramatic increase in APE1 levels during a severe ROS attack. Figure 5A–E show cell images acquired at different time points (0 min, 10 min, 20 min, 40 min, and 60 min). Five representative regions were selected and numbered, with each region marked by a differently colored box. The time courses of the fluorescence intensity of the five representative regions are shown in Figure 5F. The fluorescence intensity of background Region 5 (blue box) remains very low throughout the observation period. In contrast, the signals in Region 3 rapidly increase and reach the peak values within 10 min, reaching over 10 times higher than the brightest region shown in Figure 4E, which are observed under normal cultural conditions. This indicated a significant increase in APE1 levels in PC-12 cells following tBHP stimulation. Region 2 was in the same cell as Region 3, with fluorescence signals in Region 2 starting to increase a few minutes after those in Region 3, and the highest intensity in Region 2 being approximately half of that in Region 3. The fluorescence signals in both regions last for about 35 min, after which they return to the baseline.

Regions 1 and 4 represent the responses of two other cells in the field of view, which show distinct time courses from Regions 2 and 3, indicating different responses of different cells to the same stimulus. For Region 1, the fluorescence intensity is about 30% of that of Region 3 and only lasted for ~15 min. However, for Region 4, the fluorescence is already observed at a relatively high level at the beginning of the imaging, then the intensity declines slightly. After about 30 min, the signals start to increase again and quickly reach the maximum level. The signals last for about 25 min and then decline to the baseline. The relationship between these responses and the oxidation damage is under further investigation. Due to the high t-BHP concentration used in the treatment, significant changes in the morphology of the PC-12 cells are observed, displaying characteristics of necroptosis and apoptosis [23].

Given the significance of APE1 as a potential prognostic biomarker [25,26,27] and a therapeutic target [28,29,30], it is crucial to have easily accessible molecular tools for dynamically monitoring APE1 in vivo at the subcellular level. Our method will not only advance research on the progression of various diseases but also facilitate high-throughput screening for the discovery of anticancer drugs. Comparison between other types of normal cells and their corresponding cancerous cells merits further study.

## 3. Materials and Methods

### 3.1. Chemical Reagents and Materials

The DNA oligonucleotides were synthesized and purified by HPLC (Sangon Biotech Co., Shanghai, China). RNA strands were synthesized and purified by GenePharma Co. (Shanghai, China). The sequences of all the DNA and RNA strands that have been studied in this work are summarized in Table S1. Silica-coated magnetic Fe_3_O_4_ nanoparticles (SiMNPs) were purchased from PuriMag Biotech Co., Ltd. (Xiamen, China). Apurinic/apyrimidinic endonuclease I (APE1), Uracil-DNA Glycocasylase (UDG), Deoxyribonuclease I (DNase I), Exonuclease III (Exo III), Exonuclease I (Exo I), T5 Exonuclease (T5 Exo), T7 Exonuclease (T7 Exo) and their corresponding buffers (Table S2) were all purchased from New England Biolabs (NEB, Ipswich, MA, USA). Avidin was purchased from Sigma chemical Co. (St. Louis, MO, USA). 1-Ethyl-3-(3-dimethylaminopropyl) carbodiimide hydrochloride (EDC) and N-hydroxysulfosuccinimide sodium salt (sulfo-NHS) were purchased from Aladdin Chemical Co. (Shanghai, China). Tert-butylhydroperoxide (tBHP) was obtained from Thermo Fisher Scientific (Waltham, MA, USA).

Mammary gland epithelial adenocarcinoma cells (MCF-7) cell line, PC-12 cell line and PC-12 (highly differentiated) cell special medium (CM-0481) were purchased from Procell Life Science&Technology Co., Ltd. (Wuhan, China). Mammary gland epithelial cells (MCF-10A) cell line was purchased from iCell Bioscience Inc (Shanghai, China). Mammary Epithelial Cell Growth Medium (MEGM, CC-3150) was purchased from Lonza Bioscience (Basel, Switzerland). Dulbecco’s modified Eagle’s medium (DMEM), Dulbecco’s phosphate buffer solution without calcium & magnesium (DPBS) and F-12 Nutrient Mix were purchased from Corning (Manassas, VA, USA). Human recombinant insulin was purchased from Coolaber (Beijing, China). Hoechst 33342 was obtained from Beyotime Biotech Co., Ltd. (Shanghai, China).

### 3.2. Synthesis of DNA/RNA Hybrid Nanoprobes

In a 200 μL PCR tube, 200 pmol biotin-labeled ssDNA (D_U_, Table S1), 2 μL Uracil-DNA Glycosylase (UDG, 5000 U/mL), 2.5 μL 10 × UDG buffer and enzyme-free water was added to a total volume of 25 μL. The well-mixed solution was incubated at 37 °C for 20 min. Then, UDG was inactivated by heating it at 95 °C for 5 min to obtain a biotin-labeled AP-site containing ssDNA (D_0_). To 200 μL 4.5 μM D_0_ in 1 × DNase I buffer (10 mM Tris-HCl, pH 7.6), the avidin-modified silica-coated magnetic nanoparticles (SiMNP@AVD) were added at a final concentration of 5 mg/mL and mixed well. The solution was incubated at room temperature in a vertical shaker for 15 min. After magnetic separation and washing three times with 1 × DNase I buffer, the obtained nanoparticles were resuspended in 1 × DNase I buffer, to which RNA strands was added at 3 times the concentration of D_0_. The D_0_/R was slowly annealed from 85 °C to room temperature, followed by magnetic separation and washing to remove the excess free RNA.

### 3.3. Sensitivity and Selectivity Measurement of D_0_/R-Nanoprobes

All the measurements were carried out in 200 μL sealed PCR tubes with the total volume of the reaction solution fixed at 50 μL.

Sensitivity measurement: to the 200 μL PCR tube, 5 μL of 10 × Buffer1.1, 2 μL 0.5% Triton X-100, 5 μL of 1 mg/mL D_0_/R-nanoprobes and 36 μL of water were added and mixed well. Then, 2 μL of APE1 (final concentration 1 U/mL) was added and the detection was performed at 37 °C on a Rotor-Gene Q 5plex HRM Instrument (QIAGEN, Hilden, Germany). The fluorescence intensity of the reaction solution was monitored in real time on Rotor-Gene Q (Qiagen, Hilden, Germany). The thermal program was 250 cycles at 37 °C with 5 s per cycle, and the fluorescence was measured at the end of each cycle. Fluorescence intensity was measured once a cycle (5 s per cycle) with a gain level of 10. The excitation/emission wavelengths are 585 nm/610 nm for ROX.

Selectivity measurement: to the 200 μL PCR tube, 5 μL of 10 × Buffer1.1, 2 μL 0.5% Triton X-100, 5 μL of 1 mg/mL D_0_/R-nanoprobes, and 36 μL of water were added and mixed well. Then, 2 μL of each enzyme (final concentration is 1.0 U/mL for APE1, 1.0 U/mL for DNaseI, 2.5 U/mL for ExoI, 20 U/mL for ExoIII and 1.0 U/mL for T5 Exo, respectively) was added and the detection was performed at 37 °C on a Rotor-Gene Q 5plex HRM Instrument (QIAGEN, Hilden, Germany). The fluorescence intensity of the reaction solution was monitored in real time on Rotor-Gene Q (Qiagen, Hilden, Germany). The thermal program was 250 cycles at 37 °C with 5 s per cycle, and the fluorescence was measured at the end of each cycle. The fluorescence intensity was measured once a cycle (5 s per cycle) with a gain level of 10. The excitation/emission wavelengths are 585 nm/610 nm for ROX.

### 3.4. Intracellular Uptake of D_0_/R-Nanoprobes for Fluorescence Imaging

For PC-12 and MCF-7, the cells were incubated in the Gibco^®^ FluoroBrite™ DMEM containing the D_0_/R-nanoprobes (100 μg/mL) at 37 °C for 120 min under 5% CO_2_ on SantaiBio QY097 6–96 magnetic plate. Then, the magnetic field was removed, and the cells were washed 3–5 times with 1 × PBS to remove the extracellular magnetic beads. The nuclei were then stained with 100-fold diluted cell nuclear staining dye Hoechst 33342 in Gibco^®^ FluoroBrite™ DMEM for 5 min. After washing 3 times with 1 × PBS, Gibco^®^ FluoroBrite™ DMEM fluorescent imaging-specific medium with ultra-low fluorescent background was added for dynamic observation under a confocal microscope.

For MCF-10A cells, the incubation time was prolonged to 6 h. After the magnetic field was removed, the cells were washed 10 times with 1 × PBS to remove the extracellular magnetic beads. Other steps were performed in the same manner as described above. The Opti-MEM fluorescent imaging-specific medium was used for observation under a confocal microscope.

## 4. Conclusions

In this study, we develop a DNA/RNA hybrid-based magnetic fluorescent nanoprobe with an average size of 25 ± 3 nm, which can be efficiently taken up by various live cells using magnetic transfection. The D_0_/R-nanoprobe exhibits remarkable specificity toward APE1 and a strong resistance to cellular background interference. By utilizing this nanoprobe, we for the first time visualize the in-vivo activity of APE1 in both normal (MCF-10A) and cancerous (MCF-7) breast live cells. Additionally, we monitor the real-time changes in APE1 levels in highly metabolically active neuroendocrine cells under normal conditions and severe oxidative stress. The fluorescent nanoprobe offers a valuable tool for investigating the dynamic alterations of intracellular APE1 in normal or cancerous live cells. It also holds the potential for visible and controllable release of miRNA drugs within live cells for therapeutic purposes.

## Data Availability

Data will be available on request.

## Supplementary Materials

The following supporting information can be downloaded at: https://www.mdpi.com/article/10.3390/molecules28093935/s1, Figure S1: Zeta potential values of the silica-coated magnetic nanoparticles (SiMNP) before and after modification with avidin (SiMNP@AVD) and attachment of the biotin-labeled DNA/RNA hybrid probes; Figure S2: TEM images of the avidin-modified silica-coated magnetic nanoparticles (SiMNP@AVD) before (A) and after (B) attachment of the biotin-labeled DNA/RNA hybrid probes (Magnification: 1.2 × 10^5^); Figure S3: Uniform dispersion of D_0_/R-nanoprobes (0.1 mg/mL) in 10 mM PBS (pH = 7.4) solution (A) and rapid separation under the influence of a magnetic field (B). Figure S4: Linear calibration curve for the detection of APE1 activity by using D_0_/R-nanoprobe (0.1 mg/mL). The linear working range is from 0.01 to 1.0 U/mL and the detection limit is 0.005 U/mL; Figure S5: Fluorescence images of MCF-7 live cells acquired at 120 min after 2-h magnetic transfection of D_0_/R-nanoprobes (green). Cell nuclei were stained with Hoechst 33342 (blue); Figure S6: Fluorescence images of MCF-10A live cells acquired after 2-h magnetic transfection of D_0_/R-nanoprobes (green). Cell nuclei were stained with Hoechst 33342 (blue); Figure S7: Fluorescence images of MCF-10A live cells acquired after 6-h magnetic transfection of D_0_/R-nanoprobes (green). Cell nuclei were stained with Hoechst 33342 (blue); Figure S8: (A) Fluorescence imaging of APE1 activity (green) in MCF-10A live cells after 6 h magnetic transfection of D_0_/R-nanoprobes (50 μg/mL). (B) Fluorescence image of the MCF-10A live cells after 6 h magnetic transfection of D_U_/R-control nanoprobes (50 μg/mL, green). Hoechst 33342 was used to stain the nucleus (blue); Figure S9: Fluorescence images of PC-12 live cells acquired at 40 min after 2-h magnetic transfection of D_0_/R-nanoprobes (green). Cell nuclei were stained with Hoechst 33342 (blue); Table S1: Sequences of the oligonucleotides used in this work; Table S2: The composition and pH of the reaction buffers for the nucleases studied in this work; Table S3: Dynamic light scattering (DLS) measurement results.
