# Supplementary material for: Visualization and Comparison of the Level of Apurinic/Apyrimidinic Endonuclease 1 in Live Normal/Cancerous and Neuron Cells with a Fluorescent Nanoprobe
Source: Molecules. 2023 May 7;28(9):3935. doi: 10.3390/molecules28093935 (PMC10179877; doi:10.3390/molecules28093935)
Supplement: Supplementary file 1 [file molecules-28-03935-s001.zip › molecules-2337098-supplementary.pdf]

Supplementary Information for

Visualization and Comparison of the Level of Apurinic/Apyrimidinic Endonuclease 1 in Live Normal/Cancerous and Neuron Cells with a Fluorescent Nanoprobe

Peng Lu [1,2,†], Xiangjian Cao [1,†], Jinghui Zheng [1], Ying Sun [1], Ziyu Tang [1] and Meiping Zhao [1,*]

[1] Beijing National Laboratory for Molecular Sciences and MOE Key Laboratory of Bioorganic Chemistry and Molecular Engineering, College of Chemistry and Molecular Engineering, Peking University, Beijing 100871, China

[2] Institute of Food Science and Technology, Chinese Academy of Agricultural Sciences, Beijing 100193, China

* Correspondence: mpzhao@pku.edu.cn

[†]These authors contributed equally to this work.

Experimental Section

Zeta potential measurements

Zeta potential of aqueous solution of nanoparticles were determined using a Zeta PALS (Brookhaven Instruments Corporation, U.S.A.). The nanoparticles were dispersed in millipore water solution by ultrasonic treatment for 10 min. Approximately 0.5 mL of the obtained homogeneous solution (0.5 mg/mL) was aspirated into an EP tube. As the electrolyte, a solution of PBS (final concentration 0.01 M, pH 7.6) was then added to bring the solution volume to 2.5 mL. The obtained solution was transferred to a 4.5 cm^3 disposable polystyrene cuvette for zeta potential measurement. Blanks were prepared by substituting Millipore water for the nanoparticle solution. The measurements were performed for at least three times.

Transmission electron microscopy (TEM) imaging

Nanoparticle solutions were diluted with water to a concentration of 0.1 mg/mL and ultrasonicated for 10 min. Then a drop of the obtained solution was placed on a carbon-coated copper grid and dried in air for at least 24 h. TEM measurements were performed using a Tecnai G2 T20 Transmission Electron Microscope at an acceleration voltage of 100 kV.

Dynamic light scattering (DLS) analysis

The average hydrodynamic sizes of the nanoparticles were determined by DLS measurements using Brookhaven 90Plus Zeta (Brookhaven Instruments Corp., Holtsville, NY) at room temperature. The nanoparticles were dispersed in 10 mM PBS (pH=8.0) at a final concentration of 0.01 mg/mL through ultrasonic treatment for 20 min.

Table S1. Sequences of the oligonucleotides used in this work.

Name	Sequence[a,b]
D_u	5'-(ROX)ATCCCTGUAGATGTGAT(BHQ2)GTTGATCCTTTGGAAAAAAA-biotin
R	3'-CCUUUAGGGACCGUUACACUA[c]
D_0	5'-(ROX)ATCCCTG□AGATGTGAT(BHQ2)GTTGATCCTTTGGAAAAAAA-biotin[d]

[a] The complementary parts are underlined. The mismatched base pairs are shown in red.

[b] ROX is 6-carboxy-X-rhodamine. BHQ2 is Black Hole Quencher 2.

[c] The RNA sequence used here is the sequence of miRNA-23a.

[d] □ represents an abasic site obtained by removal of the uracil base with UDG. D_0 indicates that the ssDNA contains a □.

Table S2. The composition and pH of the reaction buffers for the nucleases studied in this work.

Name	Buffer	pH(25℃)
APE1	Buffer 1.1: 10 mM Bis-Tris-Propane-HCl, 10 mM $MgCl_2$,100 μg/mL BSA	7.0
DNase I	10 mM Tris-HCl, 2.5 mM $MgCl_2$, 0.5 mM $CaCl_2$	7.6
TREX 1	20 mM Tris-HCl, 5 mM $MgCl_2$, 2 mM Dithiothreitol	9.0
Exonuclease I	67 mM Glycine-KOH, 6.7 mM $MgCl_2$, 10 mM 2-Mercaptoethanol	9.5
T5 Exonuclease	50 mM potassium acetate, 20 mM Tris-acetate, 10 mM magnesium acetate, 1 mM Dithiothreitol	7.9

Supplemental results

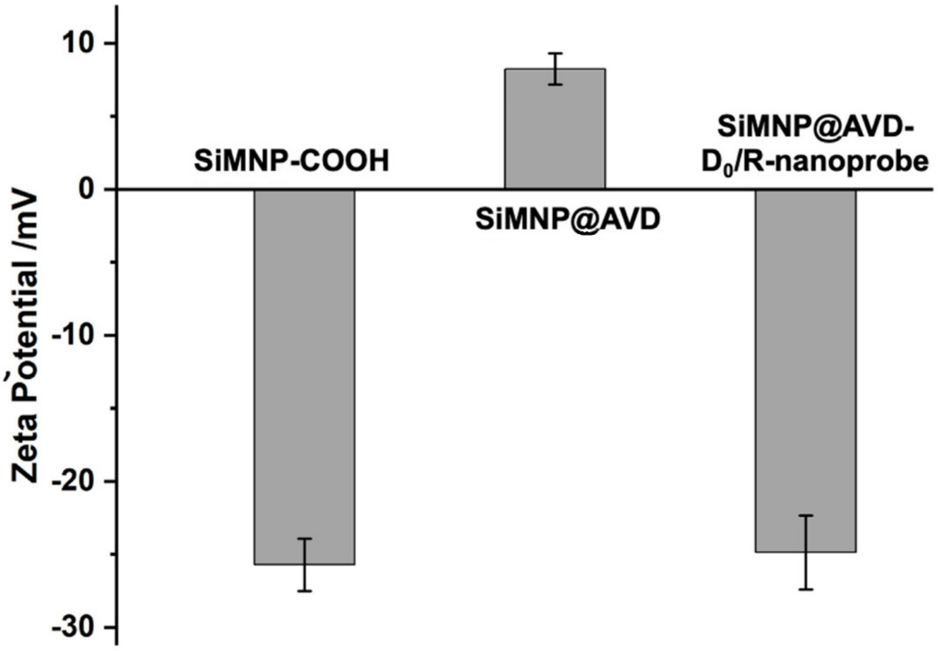

Figure S1. Zeta potential values of the silica-coated magnetic nanoparticles (SiMNP) before and after modification with avidin (SiMNP@AVD) and attachment of the biotin-labeled DNA/RNA hybrid probes.

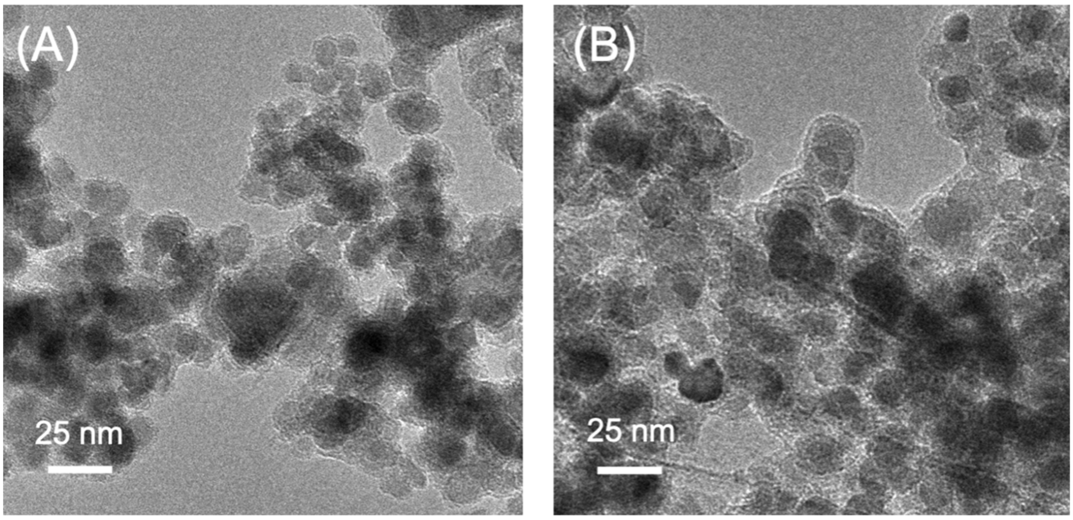

Figure S2. TEM images of the avidin-modified silica-coated magnetic nanoparticles (SiMNP@AVD) before (**A**) and after (**B**) attachment of the biotin-labeled DNA/RNA hybrid probes (Magnification: 1.2 × 10^5).

Table S3. Dynamic light scattering (DLS) measurement results.

Magnetic beads	Hydrodynamic size (nm)	Polydispersity
SiMNPs	226 ± 12	0.270 ± 0.078
SiMNP@AVD	202 ± 12	0.266 ± 0.064
SiMNP@AVD@AP-DNA/RNA (D_0/R-nanoprobe)	246 ± 16	0.219 ± 0.029

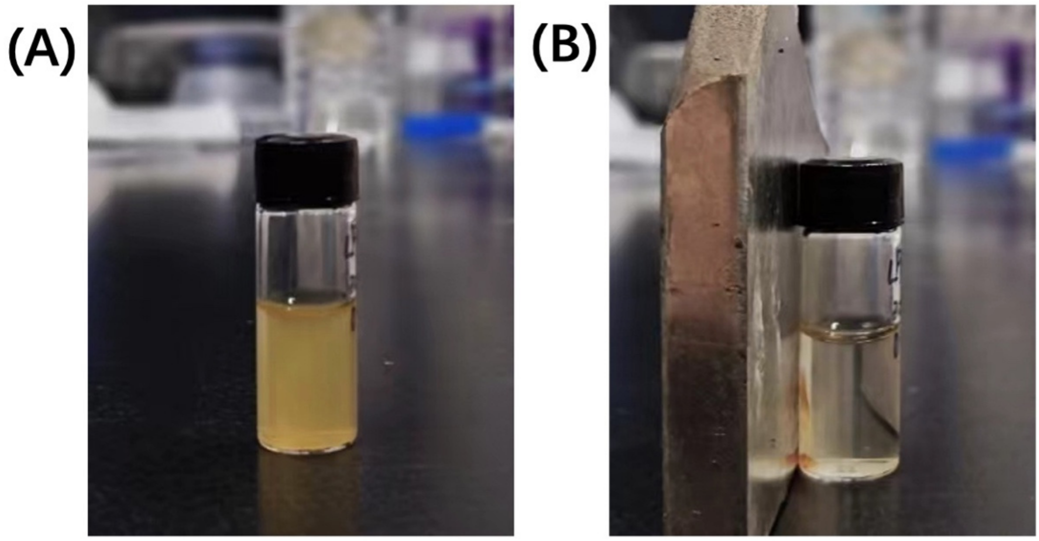

Figure S3. Uniform dispersion of D_0/R-nanoprobes (0.1 mg/mL) in 10 mM PBS (pH=7.4) solution (**A**) and rapid separation under the influence of a magnetic field (**B**).

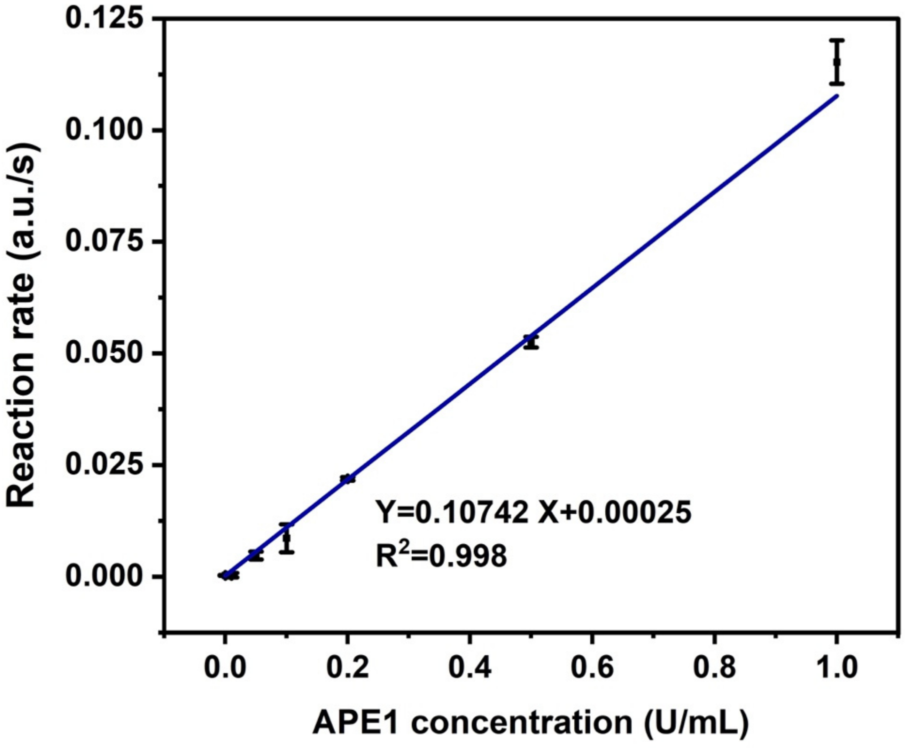

Figure S4. Linear calibration curve for the detection of APE1 activity by using D_0/R-nanoprobe (0.1 mg/mL). The linear working range is from 0.01 to 1.0 U/mL and the detection limit is 0.005 U/mL.

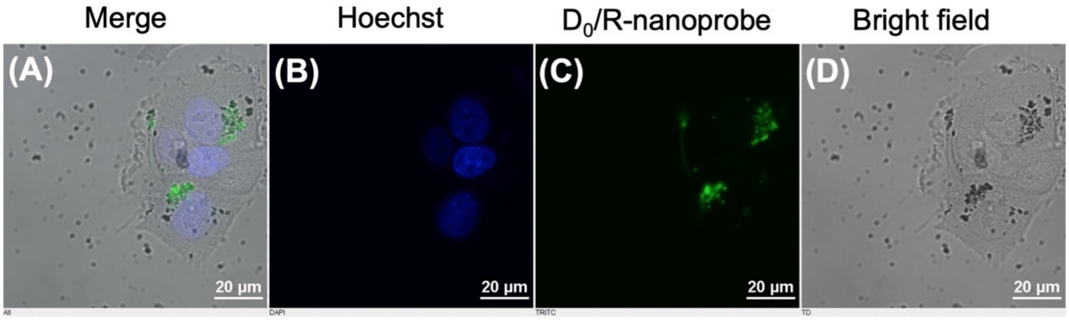

Figure S5. Fluorescence images of MCF-7 live cells acquired at 120 min after 2-h magnetic transfection of D_0/R-nanoprobes (green). Cell nuclei were stained with Hoechst 33342 (blue).

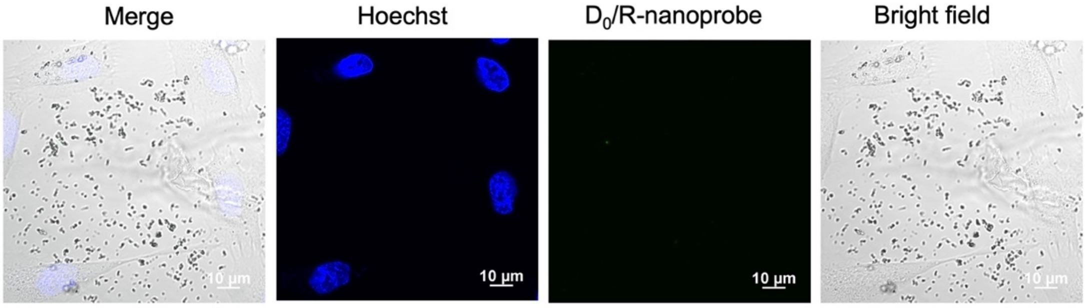

Figure S6. Fluorescence images of MCF-10A live cells acquired after 2-h magnetic transfection of D_0/R-nanoprobes (green). Cell nuclei were stained with Hoechst 33342 (blue).

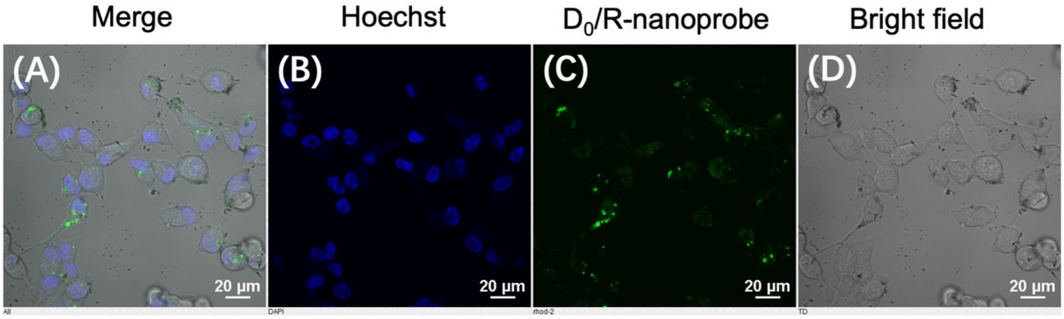

Figure S7. Fluorescence images of MCF-10A live cells acquired after 6-h magnetic transfection of D_0/R-nanoprobes (green). Cell nuclei were stained with Hoechst 33342 (blue).

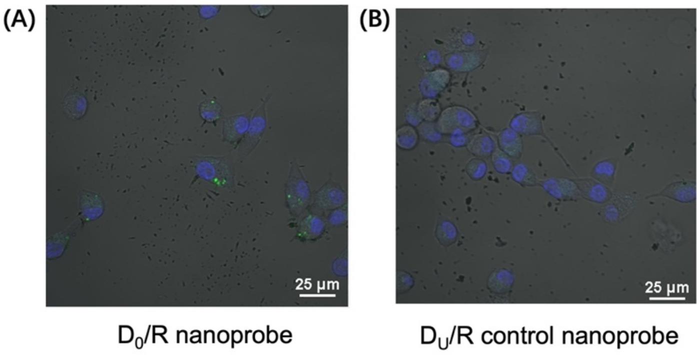

Figure S8. (**A**) Fluorescence imaging of APE1 activity (green) in MCF-10A live cells after 6 h magnetic transfection of D_0/R-nanoprobes (50 µg/mL). (**B**) Fluorescence image of the MCF-10A live cells after 6 h magnetic transfection of D_U/R-control nanoprobes (50 µg/mL, green). Hoechst 33342 was used to stain the nucleus (blue).

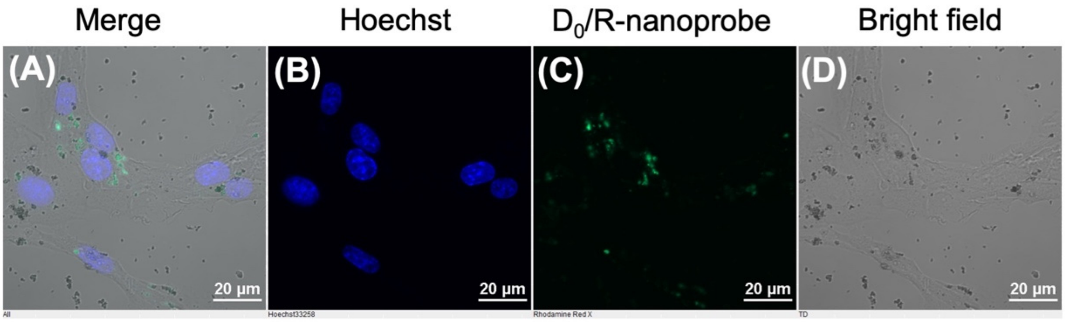

Figure S9. Fluorescence images of PC-12 live cells acquired at 40 min after 2-h magnetic transfection of D_0/R-nanoprobes (green). Cell nuclei were stained with Hoechst 33342 (blue).
